# Potential Role of *Malassezia restricta* in Pterygium Development

**DOI:** 10.3390/ijms26072976

**Published:** 2025-03-25

**Authors:** Martina Paradzik Simunovic, Marina Degoricija, Jelena Korac-Prlic, Mladen Lesin, Robert Stanic, Livia Puljak, Ivana Olujic, Josipa Marin Lovric, Ana Vucinovic, Zana Ljubic, James Thissen, Car Reen Kok, Crystal Jaing, Kajo Bucan, Janos Terzic

**Affiliations:** 1 Department of Ophthalmology, University Hospital of Split, Spinciceva 1, 21000 Split, Croatia; martina.paradzik@gmail.com (M.P.S.);; 2 Laboratory for Cancer Research, School of Medicine, University of Split, Soltanska 2, 21000 Split, Croatia; 3 Department of Medical Chemistry and Biochemistry, School of Medicine, University of Split, Soltanska 2, 21000 Split, Croatia; 4 Center for Evidence-Based Medicine and Healthcare, Catholic University of Croatia, 10000 Zagreb, Croatia; 5 Biosciences and Biotechnology Division, Lawrence Livermore National Laboratory, Livermore, CA 94550, USA

**Keywords:** pterygium, ocular microbiome, *Malassezia restricta*, *CHIT1* gene

## Abstract

Pterygium is a condition affecting the ocular surface, marked by a triangular-shaped growth of fibrotic tissue extending from the nasal conjunctiva toward the corneal center, potentially causing visual impairment. While ultraviolet (UV )light exposure is the primary risk factor for pterygium, its underlying cause remains unclear. In order to better understand the true genesis of pterygium development, we investigated pterygium tissue and compared it with healthy conjunctiva controls. Given the eye’s direct environmental exposure, we analyzed the microbiota composition using metagenomic sequencing of pterygium tissue to identify microbes potentially associated with this condition. Metagenomic sequencing revealed a higher prevalence of the fungus *Malassezia restricta* in five pterygium samples, confirmed by in situ hybridization. The *CHIT1* gene, which plays a role in antifungal defenses, displayed the highest expression in five pterygium tissue samples compared to healthy conjunctiva controls, suggesting the potential involvement of *Malassezia restricta* in pterygium development. Gene expression profiling of pterygium highlighted an IL-33 and IL-4 gene expression signature, along with an increased presence of M2 macrophages, emphasizing their role in promoting fibrosis—a hallmark feature of pterygium. The detection of *Malassezia restricta* in the pterygium samples and associated molecular changes provides novel insights into the ocular microbiome and raises the possibility of *Malassezia’s* involvement in pterygium pathology.

## 1. Introduction

Pterygium is a disease affecting the eye’s surface, characterized by irritation, aesthetic concerns, and, in the advanced stages, visual impairment. Its name is derived from the Greek *pterygos*, meaning wing, reflecting the wing-shaped tissue that extends from the nasal conjunctiva toward the center of the cornea. According to a 2018 meta-analysis, the overall prevalence of pterygium is around 12%, but the risk increases closer to the equator, suggesting that sun exposure contributes to pterygium development [1,2]. It is suggested that ultraviolet (UV) radiation disrupts the barrier separating the avascular cornea and the vascularized conjunctiva, followed by a secondary event like viral infection or additional exposure to UV rays that triggers proliferation, hyperplasia, angiogenesis, and inflammation, resulting in pterygium formation [3]. The cause of this fibrovascular lesion remains unclear; however, additional factors including dust particles, chronic irritation, and inflammation are considered to be contributing factors [2,4,5]. Surgical excision remains the only established treatment for pterygium, though recurrence rates are high, at up to 69.2% [6].

The human microbiota, a population of microorganisms that colonize various body sites, including the eye, has gained growing attention for its association with host health and disease [7]. Gut microbiota disturbances have been linked to several eye conditions, including uveitis, dry eye, age-related macular edema, and glaucoma [8,9]. While the skin and gut microbiota are well characterized, studies on the ocular surface microbiota are rather limited, even though the epithelium of the eye is directly exposed to the environment and is consequently prone to microbial colonization and infections, especially when the immune barrier is compromised [10]. Traditional culture-based microbiological studies have long suggested that the ocular surface is predominantly sterile. However, more sensitive sequencing-based techniques have identified diverse microorganisms on the eye surface [11,12].

In a study by Ozkan et al., *Pseudomonas* was identified as the predominant bacterial genus on healthy conjunctiva, with no difference between the limbal and fornix regions [13]. Similarly, Dong et al. identified 12 core bacterial genera on the healthy ocular surface and highlighted the vertical stratification of the microbiota, with variations in the findings depending on the gentle versus firm swabbing of the ocular surface [12]. Studies have also explored a potential link of pterygium with human papillomavirus (HPV), but findings from different studies vary [14]. Despite the microbiota’s rich composition, encompassing species across diverse kingdoms, most research on the ocular surface has focused on bacterial species, with a limited exploration of viruses and no reports on other components such as fungi [15].

To date, there are no definitive studies directly linking specific microbiota components to pterygium. Given the conjunctiva’s exposure to the environment and its colonization by microorganisms, it is plausible that certain microbes may play a role in pterygium formation. Therefore, this study aimed to identify and characterize the microbiota constituents potentially associated with pterygium development.

## 2. Results

### 2.1. Experimental Design and Pterygium Characteristics

**Figure 1:**
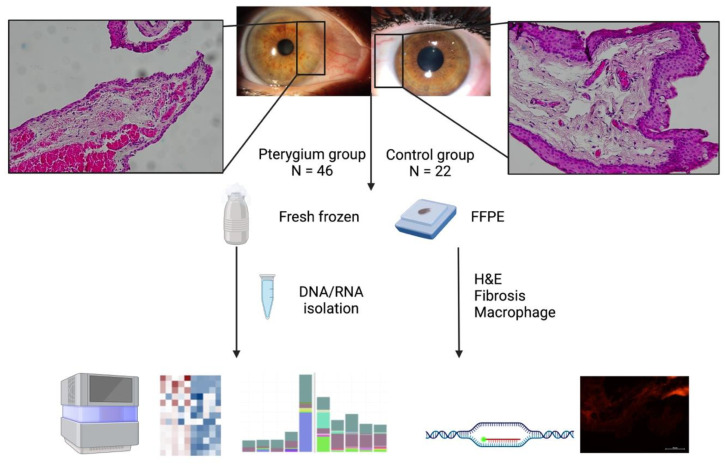
The work flow of the experiment. Created in BioRender. Paradzik Simunovic, M. (2025) https://BioRender.com/f95m049, accessed on 11 March 2025.

The flowchart of the experiment is shown in Figure 1. Hematoxylin and eosin staining of pterygium displayed both epithelial and stromal changes, squamous metaplasia with loss of goblet cells in the epithelial layer, and stroma filled with fibrovascular connective tissue consisting of collagen mixed with fibrillar structures and blood vessels with acute and chronic inflammatory infiltrate, as opposed to the control samples from the conjunctiva, where non-keratinizing squamous epithelium was presented, with scattered goblet cells covering the loose connective tissue of the lamina propria. The demographics of the patients and control subjects are presented in Table 1. There was an equal distribution of men and women in the patient and control groups. The control group was slightly older, reflecting the advanced age at which cataracts are fully developed and require surgery.

### 2.2. Metagenomic Analysis Revealed Higher Occurrence of Malassezia restricta in Pterygium Tissue, a Finding Corroborated by Fluorescent In Situ Hybridization (FISH)

**Figure 2:**
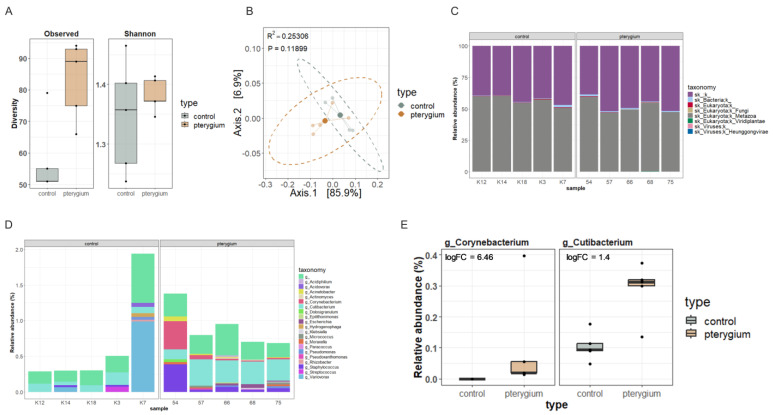
Metagenomic analysis of pterygium. Taxonomic analysis of pterygium compared with healthy conjunctiva. (**A**) Even though not statistically significant, pterygium samples have, on average, higher alpha diversity (observed species and Shannon index) compared to controls. (**B**) No significant differences in beta diversity (Bray–Curtis distance) were found between groups. (**C**) Kingdom-level relative abundance (%). (**D**,**E**) *Corynebacterium* and *Cutibacterium genera* were statistically differentially abundant between pterygium and healthy conjunctiva.

A metagenomic analysis was performed on a subset of five pterygium and five control samples (Figure 2). The taxonomic analysis of the whole shotgun metagenomic sequencing indicated no significant differences in the alpha and beta diversity, likely due to the limited sample size (Figure 2A,B). While not significant, the pterygium samples had, on average, a higher number of observed microbial species and Shannon diversity index. At the kingdom level, the samples were mainly dominated by Metazoa, with no observable differences between the patient and control groups (Figure 2C). However, the differential abundance analysis at the genus level revealed an elevated abundance of *Corynebacterium* and *Cutibacterium* in the pterygium samples, both skin commensals that are considered opportunistic pathogens (Figure 2D,E).

**Figure 3:**
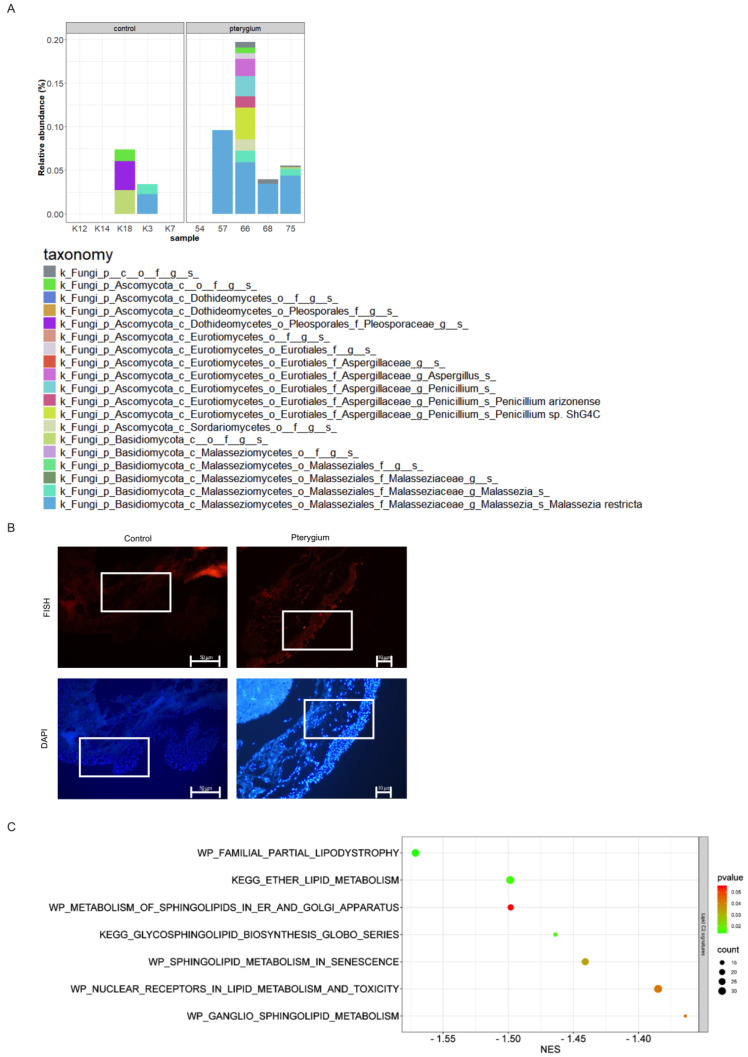
*Malassezia restricta* presence in pterygium. (**A**) Relative abundance of fungal species across pterygium and healthy control samples. (**B**) Conformation of *Malassezia restricta* with fluorescein in situ hybridization. An indicative part of the section is squared. (**C**) Lipid gene signatures.

Additionally, the pterygium samples had a higher prevalence of fungi compared to that of the healthy controls (Figure 3A). *Malassezia restricta*, in particular, was found to be present in four out of five pterygium samples, while only one of the control samples tested positive (Figure 3A). In order to further confirm the presence of *Malassezia* in the pterygium tissue, fluorescent in situ hybridization was employed (Figure 3B). Because *Malassezia species* are lipid-dependent, the lipid metabolism-related gene signatures were analyzed and found to be altered in the pterygium samples (Figure 3C).

### 2.3. Chitotriosidase (CHIT1), an Enzyme Critical for Breaking Down Chitin-Containing Pathogens like Malassezia, Showed the Highest Expression Levels in Pterygium Samples

**Figure 4:**
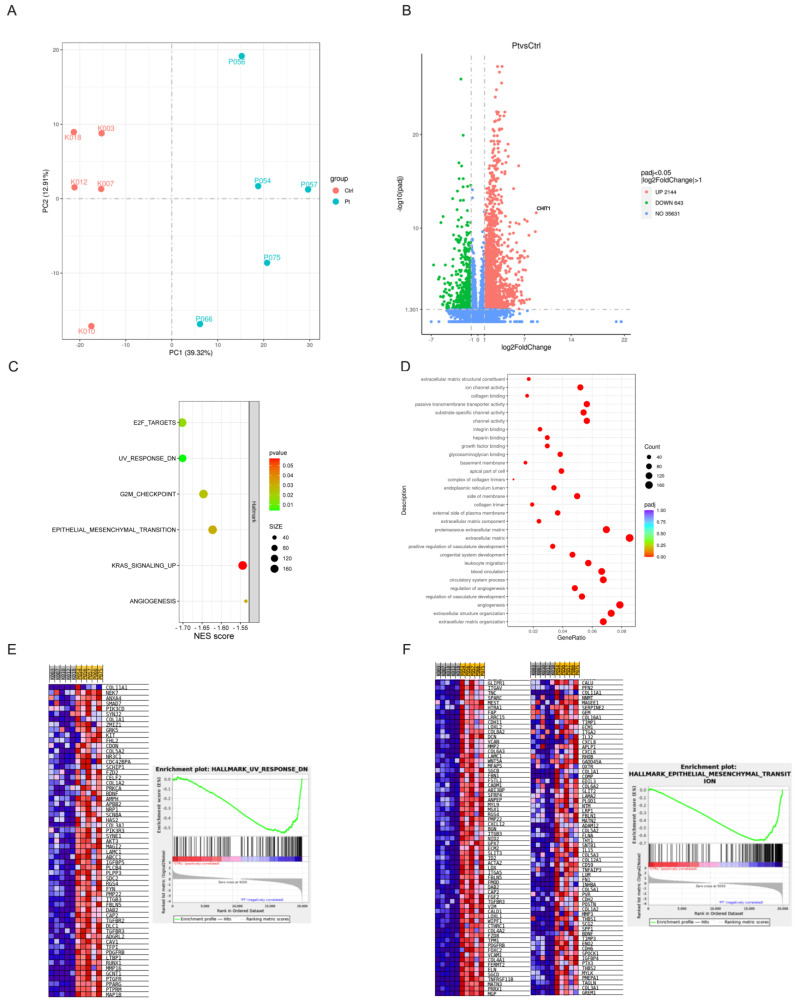
Gene expression profile of pterygium. (**A**) PCA plot. (**B**) The differentially expressed genes between pterygium and controls visualized in volcano plot. (**C**) Gene ontology. (**D**) HALLMARK gene signature GSEA. (**E**) UV response in HALLMARK gene signature. (**F**) Epithelial–mesenchymal transition in HALLMARK gene signature. Hallmark gene sets enriched in Pterygium phenotype. FDR < 25% total of 6.

Our transcriptomic analysis revealed significant differences in gene expression between the pterygium and control samples (Figure 4). A principal component analysis (PCA) and a volcano plot, performed to distinguish most of the variation in gene expression, showed a good correlation and good quality of the obtained transcriptome data (Figure 4A,B). In the gene ontology (GO) analysis, we recognized several biological processes that were significantly enriched in the pterygium (Figure 4C). Among the upregulated, differentially expressed genes, the highest increase in expression was found for the *CHIT1* gene, which is involved in the degradation of chitin, a component of the *Malassezia* cell wall. The upregulation of this gene was followed by that of genes involved in the extracellular matrix structure, *COL10A1*, and remodeling, *MMP9*, reflecting intensive fibrosis, the main pathological finding of pterygium. Angiogenesis, a well-known characteristic of pterygium, and regulation of the vascular component are also biological processes upregulated in pterygium. The GO classification indicates the upregulation of molecular processes associated with channel activity in pterygium (Figure 4C). To further investigate the differences in their biological functions, we analyzed the HALLMARK gene set enrichment analysis (GSEA), where we found the following upregulated pathways: E2F transcription factor, UV response, EMT, K RAS signaling pathway, and angiogenesis (Figure 4D). Additionally, genes in the UV response and EMT pathways are shown in the corresponding heatmaps, confirming the strong activation of the corresponding processes. (Figure 4E,F).

### 2.4. Fibrosis and an Immunosuppressive State Enriched with M2 Macrophages—A Hallmark of Malassezia-Associated Infections—Were Present in the Pterygium Microenvironment

**Figure 5:**
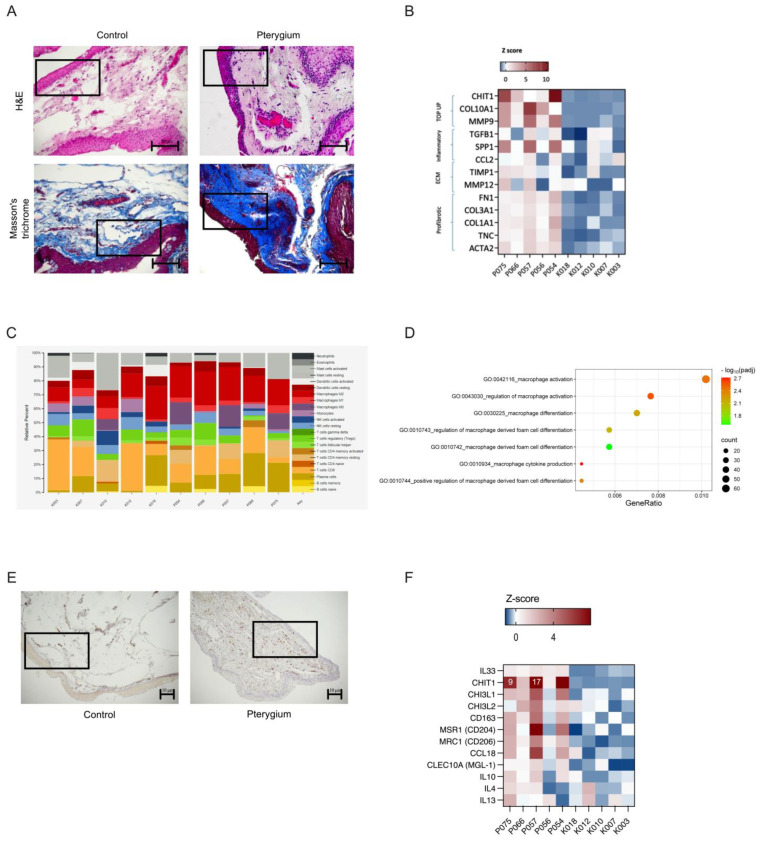
Alternately activated M2 macrophages in pterygium. (**A**) Fibrosis. (**B**) Inflammation and fibrosis. (**C**) Macrophage CD staining. (**D**) IL33 signature. (**E**) Immunohistochemical staining for a marker of M2 macrophage CD163 was present in pterygium and almost absent from control tissues. (**F**) Genes involved in alternative activation of macrophages. An indicative part of the section is squared.

An extensive fibrotic process in the pterygium tissues was observed after Masson’s trichrome staining (Figure 5A). Figure 5B demonstrates the elevated expression of genes involved in extracellular matrix turnover. The highest rise in gene expression in pterygium was observed for chitotriosidase (*CHIT1*), followed by two extracellular matrix-related proteins—collagen type X alpha one chain (*COL10A1*), and matrix metallopeptidase nine (*MMP9*). Other extracellular matrix remodeling enzymes were upregulated, including *TIMP1* and *MMP12*, as well as profibrotic genes like collagen, fibronectin, and actin genes. Fibrosis is a process facilitated by alternatively activated (M2) macrophages. Thus, we tested for the presence of M2 macrophages in the pterygium samples. The M2 gene expression signature was found to be prevalent among the pterygium samples (Figure 5C). To additionally confirm the involvement of alternatively activated macrophages in pterygium, immunohistochemical staining for CD163, a marker of M2 macrophages, was performed. Numerous activated M2 macrophages were present in the pterygium samples, while they were almost absent from the control tissues (Figure 5E). Significantly upregulated macrophage gene signatures, as well as the prominent expression of IL33 and specific markers such as *CHIT1*, *MRC1*, *CCL18*, *IL10*, and others, indicate the alternative activation of M2 macrophages (Figure 5D,F).

## 3. Discussion

In this study, we analyzed the microbiome of pterygium samples and compared it to that of healthy conjunctival tissues. We identified a notable fungal abundance in most of the pterygium samples, with the prevalence of *Malassezia restricta*. Thus, we propose a potential association between *Malassezia restricta* and the development of pterygium.

Various body niches contain diverse microbiota, and alterations in the microbiota, dysbiosis, have been implicated in the pathogenesis of several diseases [16,17]. The ocular surface was historically described as a microorganism-free environment. However, recent studies have identified it as a site with significant microbial diversity [18]. For instance, previously, a DNA-based analysis of healthy ocular surface microbiota, using conjunctival swabs, found 12 core bacterial genera, including *Pseudomonas*, *Propionibacterium*, *Bradyrhizobium*, *Corynebacterium*, *Acinetobacter*, *Brevundimonas*, *Staphylococci*, *Aquabacterium*, *Sphingomonas*, *Streptococcus*, *Streptophyta*, and *Methylobacterium* [12]. However, non-bacterial components of the conjunctiva such as viruses, fungi, archaea, and protists, remain largely unexplored. To address this gap, we undertook a metagenomic analysis of both healthy conjunctival and pterygium samples to gain insight into their microbiota composition. Surprisingly, we found *Malassezia restricta* to be present in four out of five of the tested pterygium samples. This unexpected finding prompted us to conduct an additional analysis to gain a deeper understanding of this association.

*Malassezia* species have been identified as the causative agent in certain skin conditions, making them a good candidate to be involved in pterygium development [19]. Moreover, recent studies have linked *Malassezia* with other pathologies like pancreatic ductal adenocarcinoma (PDAC), Crohn’s disease, and exacerbation of lung conditions in cystic fibrosis (CF) patients [20,21,22,23]. These findings suggest that *Malassezia* can act as a pathogen in different tissues under favorable local conditions [21]. The most detailed understanding of the mechanism by which *Malassezia* causes a disorder is described for PDAC. PDAC cells release IL-33 upon stimulation by *Malassezia.* Inteleukin-33 then promotes a local type 2 immune response characterized by the presence of Th2 and innate lymphoid cells 2 (ILC2). Such an immune milieu supports PDAC growth [24]. Interleukin-33 also triggers Th2 and ILC2 to release IL-4 and IL-13, cytokines that drive the differentiation of macrophages into an alternative, M2-like phenotype [25]. Those types of macrophages resolve inflammation, promote wound healing, and induce fibrosis [26,27]. We determined a high abundance of M2-polarized macrophages in pterygium tissues by immunohistochemical analysis and gene expression profiling. Alternatively activated, M2 macrophages are considered to be a major driver of fibrosis, a process typical for pterygium, which was also found to dominate histologic and gene expression findings in our study. The major molecular driver of fibrosis is TGF β1 produced by macrophages [28]. TGF β1 was found to be elevated in the pterygium tissue in our study, contributing to possible *Malassezia* involvement in pterygium development via the interplay of immune milieu stimulated by *Malassezia*, the immune response, the activation of macrophages, and, finally, fibrosis.

Supporting evidence for the potential involvement of *Malassezia* in pterygium is emphasized by the observation that the most notable change in gene expression occurred in the CHIT1 gene, which encodes chitinase 1. Chitinase 1 is an enzyme secreted by activated human macrophages, playing a protective role via the degradation of chitin, a key component of the cell walls of *Malassezia* [29,30]. The increased expression of CHIT1 indicates an innate immune reaction targeting *Malassezia restricta*. Moreover, *Malassezia* is a lipid-dependent yeast, and we revealed the altered expression of genes involved in lipid metabolism in the pterygium samples. This finding suggests that *Malassezia* may actively contribute to the development of pterygium rather than merely being coincidentally present [31,32].

Interestingly, we found gene expression signatures indicative of UV exposure, angiogenesis, the epithelial–mesenchymal transition, and proliferation; hallmarks well recognized in pterygium [33]. Shotgun metagenomic sequencing also revealed the presence of *Cutibacterium*, a genus of resident commensal skin bacteria, in our pterygium samples. However, this bacterial genus is typically associated with a pro-inflammatory response, a characteristic mainly absent in our pterygium samples [34].

This study has some limitations. First, the smaller sample size of the metagenomic analysis might influence the generalizability of our results. Finally, the cross-sectional and single-center design of the study might also affect our results.

By exploring a low-biomass niche like the eye surface and using a novel amplification-based methodology such as polymerase chain reaction and shotgun metagenomic sequencing, new findings about microbiota are coming to light. Fungal infections of the eye encompass a broad range of fungal diseases affecting the adnexa (eyelid, conjunctiva, and lacrimal system), orbit, and intraocular structures, varying from mild conditions to severe infections linked with systemic involvement. Fungal keratitis presents with a wide range of clinical findings, which can make diagnosis and management difficult. Recently, metagenomic deep sequencing was proposed as a quick diagnosis tool that ensures accuracy, enhancing the precision of fungal corneal diagnosis without the need to wait for fungal growth [35]. In contrast to fungal keratitis, which represents 30–50% of all microbial keratitis cases in developing countries, fungal conjunctivitis is a rare condition, primarily due to its low incidence and nonspecific clinical symptoms [36].

It is likely that the microbiome contributes to the physiology and pathology of ocular surface. However, the true clinical implication of the microbiome’s influence on pterygium needs to be fully answered.

## 4. Materials and Methods

### 4.1. Informed Consent

Written informed consent was obtained from all patients and control participants for sample collection and following analysis.

### 4.2. Patients

We studied 46 patients undergoing primary pterygium surgery who were referred to the Department of Ophthalmology, University Hospital of Split, from September 2017 to January 2019. According to the study protocol, the inclusion criteria were a pterygium larger than 4 mm on the cornea with symptoms related to visual disturbance, chronic inflammation, and cosmetic reasons [37]. Exclusion criteria were any other eye disease with (e.g., glaucoma disease) or without the use of eye drops or previous eye surgery. Control samples (n = 22) were obtained from patients who underwent cataract surgery from September 2021 to January 2022 following the procedure described in the literature [38].

### 4.3. Tissue Samples

The surgical draping was made in the standard fashion. The samples of excised pterygium tissue overlaying cornea and control samples of healthy conjunctival tissue near corneal–scleral junction were taken at the very beginning of the surgery to avoid contamination and excessive manipulation of the tissue. The collected samples were placed in sterile Eppendorf tubes, snap-frozen in liquid nitrogen, and stored at −80 °C until isolation.

### 4.4. Histological and Staining Analysis

Tissue samples were fixed in 10% buffered formalin and routinely embedded in paraffin sections. Sections of 4 mm thickness cut by microtome (RM2125 RTS, Leica, Buffalo Grove, IL, USA) were advanced to hematoxylin and eosin staining (Sigma-Aldrich, Darmstadt, Germany and Merck, Darmstadt, Germany) or Masson’s trichrome staining (BioGnost Ltd., Zagreb, Croatia).

### 4.5. Immunohistochemistry

Immunohistochemistry analysis was performed on 4 mm thick sections. After rehydration, sections were boiled in 1 mM pH 8 EDTA buffer for 30 min followed by blocking in 1% BSA/TBS-T. Anti-CD163 antibody (Abcam, ab182422, 1:500, Cambridge, UK) was used for detection of CD163+ M2 macrophages.

### 4.6. FISH

In order to visualize *Malassezia* in collected specimens, fluorescent in situ hybridization was performed using fluorescence-labeled fungi-specific oligonucleotide probe [Cy3] CCG ATA TTT AGC TTT AGA TGG AGT CTA, as described previously [39]. Microscopical examination was performed using an Olympus BX43 (Olympus Corporation, Tokyo, Japan).

### 4.7. DNA and RNA Extraction

Tissues were homogenized with Minilys system and RNA and DNA were purified using Trizol reagent according to manufacturer’s protocol and dissolved in water. Concentration of RNA and DNA were quantified using Nanodrop.

### 4.8. RNA Sequencing

To investigate the full spectrum of gene changes involved in pterygium, we used the RNA sequencing approach. For transcriptome analysis, total RNA from five representative pterygium and five control tissues was enriched for mRNA with poly-T oligo magnetic beads, and fragmented and hexamer primers were used for first-strand cDNA synthesis. A non-directional library was constructed and samples were sequenced with 40M reads per sample on Illumina Novaseq PE150 (Illumina, Inc., San Diego, CA 92122, USA). Deseq2 was used for differential gene analysis and log2FC≥ from ±1 was used as the threshold for differential expression [40].

GenePattern GSEA module (version 20.4.0) was used for gene set enrichment analysis, with one thousand permutations per gene set on collections from Molecular Signatures Database. CIBERSORTx deconvolution algorithm was used to predict relative proportions of immune cells in the tissue [41].

### 4.9. Whole-Genome Microbiome Sequencing

Genomic DNA samples were quantified using a Qubit fluorimeter (Thermo Fisher, Waltham, MA, USA) and 150 nanograms of genomic DNA of each sample was input into the Illumina DNA Prep (Illumina, #20060059, San Diego, CA, USA) library preparation kit following the standard manufacturer’s protocols. Completed libraries were assessed and quantified using a Tapestation 4200 instrument (Agilent, Santa Clara, CA, USA). Libraries were normalized and sequenced on an Illumina NextSeq 2000 sequencer using a P2 300 cycle sequencing kit (Illumina, Inc., San Diego, CA 92122, USA).

Following sequencing, fastq files were generated and quality-filtered with fastp using the following non-default parameters: -g --poly_g_min_len = 5 -x --poly_x_min_len = 5 -y [42,43]. Filtered fastq files were used as input for Centrifuge, a bioinformatic pipeline for taxonomic classification of metagenomic sequences, using a custom database that was built on the contents of NCBI nt as of 4 January 2023. The Centrifuge output was generated using a minimum hit length (MHL) of 15 and was input into Recentrifuge for further comparative analysis of the metagenomic data at an MHL of 40 [44].

### 4.10. Statistical Analysis

Biostatistical microbiome analysis and visualization were conducted in R (version 4.2). Recentrifuge counts were imported into phyloseq [45] and alpha and beta diversity measurements and taxonomic relative abundances were obtained. Wilcoxon rank-sum tests were used for between-group alpha diversity (observed species and Shannon index) comparisons. Compositional differences between groups as represented by Bray–Curtis distances were determined using PERMANOVA analysis with the vegan package [46]. Differential abundance analysis of taxonomic data was carried out using DESeq2 [40].

## 5. Conclusions

In conclusion, we identified an association between *Malassezia restricta* and pterygium. Several lines of evidence, including the highest expression of CHIT1, high prevalence of M2 macrophages, and elevated expression of IL33, suggest that this yeast could actively contribute to the development of pterygium rather than being a coincidental finding. However, further clinical studies are needed to determine whether *Malassezia restricta* is causative or one of several contributing factors associated with pterygium development.

## Figures and Tables

**Table 1:** Baseline characteristics of the subjects enrolled in the study.

Parameter	Pterygium Group (N = 46)	Control Group (N = 22)	*p*
Sex—N (%)			
Male	23 (50%)	9 (41%)	0.482
Female	23 (50%)	13 (59%)	
Age (yr)	64.3 (36.48–87.69)	71.64 (56.05–83.31)	0.015

Group comparisons were performed using the *t* test or χ^2^ test, and the significance level (*p*) was set to <0.05.

## Data Availability

All data and materials are available upon request due to GDPR restrictions.
